# Validation of the Japanese Version of the Center for Epidemiologic Studies Depression Scale—Revised: A Preliminary Analysis

**DOI:** 10.3390/bs11080107

**Published:** 2021-07-24

**Authors:** Hirohito Tsuboi, Yui Takakura, Hiromasa Tsujiguchi, Sakae Miyagi, Keita Suzuki, Thao Thi Thu Nguyen, Kim Oanh Pham, Yukari Shimizu, Yasuhiro Kambayashi, Naoko Yoshida, Akinori Hara, Hiroyuki Nakamura

**Affiliations:** 1 Institute of Medical, Pharmaceutical and Health Sciences, Kanazawa University, Kanazawa 920-1192, Japan; ytakakura@stu.kanazawa-u.ac.jp (Y.T.); naoko@p.kanazawa-u.ac.jp (N.Y.); 2 Department of Hygiene and Public Health, Graduate School of Medicine, Kanazawa University, Kanazawa 920-8640, Japan; t-hiromasa@med.kanazawa-u.ac.jp (H.T.); keitasuzuk@stu.kanazawa-u.ac.jp (K.S.); ahara@m-kanazawa.jp (A.H.); hnakamu@staff.kanazawa-u.ac.jp (H.N.); 3 Innovative Clinical Research Center, Kanazawa University, 13-1 Takaramachi, Kanazawa 920-8640, Japan; smiyagi@staff.kanazawa-u.ac.jp; 4 Department of Epidemiology, Faculty of Public Health, Haiphong University of Medicine and Pharmacy, 72A Nguyen Binh Khiem, Ngo Quyen, Hai Phong 042-12, Vietnam; toi_fs@yahoo.com; 5 Department of Environmental and Preventive Medicine, Graduate School of Medical Science, Kanazawa University, 1-13 Takaramachi, Kanazawa 920-8640, Japan; kimoanhpham129@gmail.com; 6 Department of Nursing, Faculty of Health Sciences, Komatsu University, 1-14 Mukaimotoorimachi, Komatsu 923-0961, Japan; yukari.shimizu@komatsu-u.ac.jp; 7 Department of Public Health, Faculty of Veterinary Medicine, Okayama University of Science, 1-3 Ikoinooka, Imabari 794-8555, Japan; y-kambayashi@vet.ous.ac.jp

**Keywords:** CES-D, CESD-R, depressive symptoms, epidemiological study, Japanese version

## Abstract

To make the Japanese version of the CESD-R—a revised version of the Center for Epidemiologic Studies depression scale (CES-D)—in the assessment of depressive symptoms in a general population. The English version of CESD-R was translated into Japanese, and back-translated into English by three native speakers of Japanese and English; then, we selected the version most completely consistent with the original items. The CESD-R was applied to 398 community-dwelling people (191 men: 48.0%, and 207 women: 52.0%) who were over 40 years old. The Japanese version of the CES-D was also carried out in the same population. Factor analysis was performed. Additionally, the correlations between the CESD-R and CES-D results were identified. The CESD-R scores showed a significantly positive correlation with CES-D scores (*r* = 0.74, *p* < 0.0005). Analysis of the CESD-R yielded a Cronbach’s alpha result of 0.90. Factor analysis revealed one principal factor in the CESD-R, whereas the original CES-D had two factors because of reversed items. The Japanese version of the CESD-R appears to have the reliability to be applicable for assessing depressive symptoms in population-based samples. However, because the Japanese expressions for some items might be unusual, our study population was also limited; further studies on other populations and on incorporating improved Japanese terminology will be needed.

## 1. Introduction

Depression is a common illness worldwide, with more than 264 million people affected, and it is a major contributor to the overall global burden [1]. It is one of the most common, costly, and heterogeneous mental disorders, associated with decreased social functioning and increased mortality [2]. The 12-month prevalence of major depressive disorder (MDD) in Japan is 2.7%, and the lifetime prevalence of it is 5.7% [3]. These figures are lower than those of high-income countries, including the US (8.3%), France (5.9%), and Germany (3.0%) [2], although the figures may be affected by cultural differences [4], or due to different methodological factors of various studies [2]. MDD and what are merely depressive symptoms should be distinguished [5], as indicated by the fact that depressive symptoms among university students assessed by the Beck Depression Inventory (BDI)-II [6] were higher in Japan in comparison with Western countries [7]. However, assessing depressive symptoms and the prevention of depression is crucial, since depression is associated with an increased risk of mortality in general community populations, as well as in patient populations with chronic illnesses [8]. Furthermore, over the last few years, the coronavirus disease 2019 (COVID-19) pandemic increased the rates of probable depression to two to nine times higher during the second wave than before the COVID-19 pandemic in Japan [9]. The COVID-19 pandemic is also a major problem for people’s psychological status all over the world, as mentioned in several review articles [10,11]. An accurate assessment of depression severity has become ever more important for its management in the general population.

The Center for Epidemiologic Studies Depression (CES-D) scale has been one of the most widespread scales for assessing depression since it was published in 1977; it was originally devised for screening and research in general population epidemiological studies and primary care [12]. However, more than forty years have passed since its publication, which necessitated the creation of the CES-D—Revised (CESD-R) scale. The CESD-R is an updated version of the CES-D that closely reflects the Diagnostic and Statistical Manual of Mental Disorders (DSM)-IV criteria for depression [13]. Because the CES-D does not reflect current diagnostic criteria for depression and depressive symptomatology, as defined by the DSM-IV [14], a revision of the CES-D was undertaken to indicate general dysphoria and reflect the nine primary symptoms of a major depressive episode, according to DSM-IV or -5, more reliably [15]. The Japanese version of the CES-D was validated using the original CES-D scale and was published in 1985 [16]. It was in force until recently without being updated. The CESD-R was translated into Arabic, Bhutanese, Chinese, Croatian, French, German, Korean, Latvian, Lithuanian, Polish, Portuguese, Spanish, and Turkish [17]. The Arabic, Indonesian and Polish versions appeared to be well validated [18,19,20]. Simultaneously, studies using the Spanish version were published [21,22].

There is yet to be a Japanese version of the CESD-R for which its reliability and validity have been confirmed. Thus, the aims of the present study were to translate the English version of CESD-R into Japanese, explore its psychometric properties, and establish preliminary norms. Additionally, the characteristics of CESD-R were investigated by comparing the results with the CES-D.

## 2. Participants and Methods

### 2.1. CESD-R Questionnaire

The CESD-R scale is a self-administered scale consisting of 20 statements. In relation to each statement describing well-being or behavior, the subjects choose one of five possible answers regarding their frequency. Answers from 0 (not present at all, or less than 1 day) to 4 (occurs almost every day for 2 weeks) are added together. Based on the result, the severity of depression in an individual can be determined. The lowest possible score is 0 points, the highest, 80 points (Table S1).

### 2.2. Translation Procedure

The Japanese version of CES-D was validated [16], whereas CESD-R has not yet been translated into Japanese, nor validated. The CESD-R was translated into Japanese with the permission of the author (the scale is not copyrighted) [13]. In the beginning, three native Japanese speakers independently translated the 20 English test items into Japanese. Subsequently, three native speakers of English back-translated them from Japanese into English. Then, we selected a back-translated item that was the one we considered the most completely consistent with the original version for each item, both in terms of instructions and individual test items. Finally, an English philologist was asked for an additional assessment of all translations (original scale, Japanese translation, and back-translation), and the final wordings of the Japanese version of the CESD-R scale were determined.

### 2.3. Study Population

The participants were 398 community-dwelling people of 41–90 years old (mean age ± S.D.: 62.0 ± 10.36) of the “Shika study” project of 2019–2020, which has been carried out on the Noto Peninsula, Ishikawa, Japan since 2011. This project aims to find developed solutions for lifestyle diseases by investigating community-dwelling people aged 40 years or older. All respondents were literate, understood the Japanese language well, and were requested not to use proxy respondents. The study protocol and informed consent procedure were approved by the Ethical Committee at Kanazawa University (on 18 December 2013, receipt number 1491). Written informed consent was obtained in all cases.

### 2.4. Study Procedures

The survey was performed as follows. A self-administrated questionnaire that included CES-D and CESD-R in addition to the demographic measures was distributed to the participants beforehand and collected on the examination day. In case the questionnaire had some blank spaces, the research staff supplemented it by asking questions of each participant on the day of the examination. The entire process was conducted with careful attention to the attendees’ privacy. Each item of the CESD-R was presented in Table S2.

### 2.5. Analysis

Data were analyzed using the Japanese version of SPSS Statistics version 25 (IBM Japan, Tokyo, Japan). *T*-test was used for the comparison of data between men and women. For the correlation test, age and BMI were controlled, and a *p*-value < 0.05 was set as significant. Next, we applied factor analysis to identify the underlying factor structure of CES-D and CESD-R using the Cronbach’s alpha, Kaiser–Meyer–Olkin (KMO) and Bartlett sphericity tests.

## 3. Results

### 3.1. Scale Properties of CES-D and CESD-R

As shown in Table 1, the average scores ± S.D. of CES-D and CESD-R among all responders were 10.9 ± 6.63 and 4.3 ± 6.52, respectively. The internal consistencies of CES-D (after inverting the reversed items numbered 4, 8, 12, and 16) and CESD-R were reasonably high (Cronbach’s alpha: 0.84 and 0.90, KMO: 0.91 and 0.89, Bartlett sphericity test: χ^2^ = 2900 at *p* < 0.0005, and χ^2^ = 3896 at *p* < 0.0005, respectively). There were no sex differences in CES-D (men 10.8 ± 6.34, women 11.1 ± 6.90) and CESD-R scores (men 4.3 ± 6.37, women 4.3 ± 6.67) (*t*-test: *t* = 0.52, *p* = 0.60; *t* = 0.01, *p* = 0.99, respectively).

**Figure 1:**
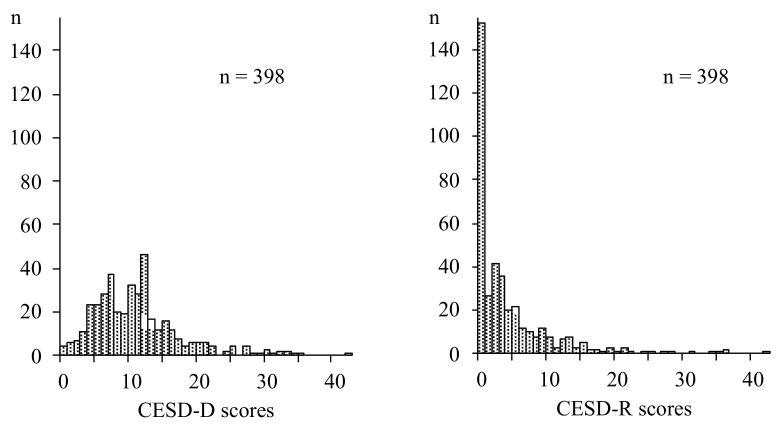
Distributions of CES-D and CESD-R results displayed as histograms of CES-D and CESD-R results, expressed by every two scores. (*n* = 398, *n* = 398, respectively).

**Figure 2:**
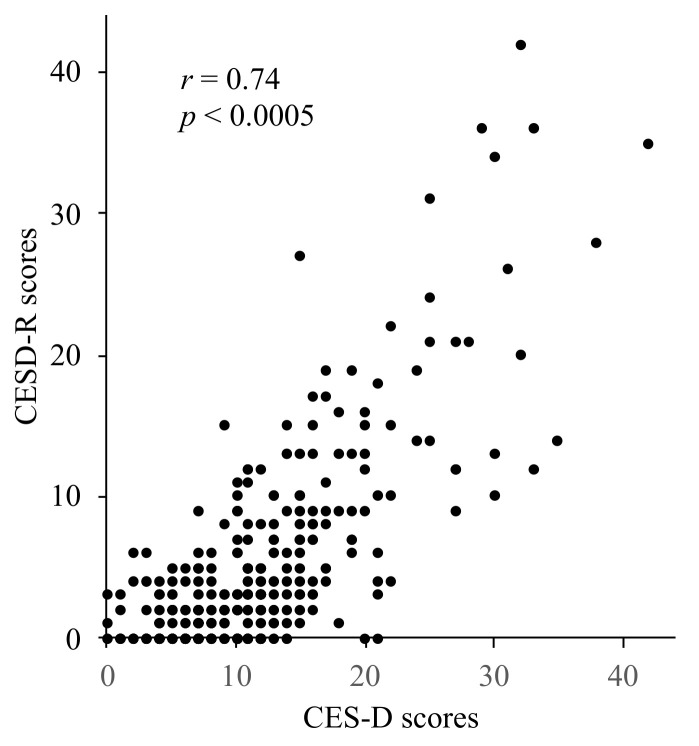
Correlations between CES-D and CESD-R scores. The total scores of CESD-R were significantly positively correlated with those of CES-D after controlling for age and BMI (*r* = 0.74, *p* < 0.0005). BMI: body mass index.

Figure 1 shows the distribution of the CES-D and CESD-R scales, and Figure 2 displays correlations between the CES-D and CESD-R scores. Both scale scores were significantly positively correlated with each other (*r* = 0.74, *p* < 0.0005).

### 3.2. Factor Analysis

In order to determine a factor structure for our sample, we employed a maximum likelihood factor analysis, followed by a promax oblique rotation for CES-D, to obtain a final solution. The number of factors was determined by the point at which the slope of the scree plot curve was clearly leveling off [23]; consequently, factor analysis extracted one factor for the CESD-R, and two factors for the CES-D (Table 2). Although factor loadings of items 1, 11, 15 and 18 of the CESD-R were low, there were no appropriate categories, even with extracting two or more factors by lowering the eigenvalue. Factor 1 in the CESD-R can explain depressive symptoms. Factors 1 and 2 in CES-D can mean depressive symptoms and positive affect, respectively.

## 4. Discussion

We translated the original English version of the CESD-R scale into Japanese and investigated the Japanese adaptation for testing depression levels in community-dwelling people. The translated version was identified to have one factor that flagged “depression” and high internal consistency. However, factor loadings of some items were not high, which might not necessarily result in establishing preliminary norms.

### 4.1. CESD-R Scores

The average CESD-R scores ± S.D. in the present study were 4.3 ± 6.52, with no significant differences between men and women. The average appeared to be a little lower in comparison with that found in other studies. For example, an original paper validating CESD-R showed 10.3 ± 11.7 on the internet survey, and 16.4 ± 13.5 among undergraduate psychology students [13]. Further, the average CESD-R scores among university students of the United States (US) were 10.2 ± 8.74 [24]. Another study with 98 African Americans and one white respondent, with an average age of 59 years old, in Washington DC, US, showed 5.8 ± 8.88 [25]. Unfortunately, we did not find the average or distribution of CESD-R scores among a similar population to the present study in published manuscripts, even though validations were carried out in Arabic, Indonesian, and other languages [18,19]. However, studies using other questionnaires indicated that the extent of depressive symptoms varies in populations, and the depressive degrees among community-dwellers in rural areas such as those in the present study were lower [26,27].

### 4.2. Factor Loadings of CESD-R

Factor analysis revealed that the CESD-R had one factor. The factor loading of some CESD-R items was not particularly high (Table 2). The reason for the low factor loadings for certain items was that the answers were biased toward the asymptomatic. These items (items 1, 5, 9, 11, 12, 14, 15, 18, 19) are questions asking whether respondents have signs of severe depression [28], such as physical symptoms, insomnia or cognitive impairment, or suicidal ideation. The participants of the current study were community-dwelling adults living in the countryside, including one MDD patient. For example, the CES-D scores of people in a rural area were lower in comparison with those in an urban area [29]. Thus, assessment among other populations will be needed.

### 4.3. Factor Structure of CES-D

CES-D consisted of two factors: depression and positive affect. However, there were problems in four reverse-scored items (items 4, 8, 12, 16) that evaluated positive affect. There were 27 participants with CES-D scores that equaled 12 who selected “0” in all items, including the four reverse-scored items. We suspect that those participants must have selected “3” in the four reversed items. It appears to be common that reverse-scored items are associated with measurement problems and cluster into a separate factor [30]. The scores of the reverse-scored items can make the CES-D distribution slightly biased to the right. In this regard, a CESD-R without any reverse-scored items may be more suitable for evaluation.

### 4.4. Comparison of CESD-R and CES-D

It should be noted why the average score in the CESD-R is approximately half as high as those in the CES-D. The CESD-R was a 5-point Likert scale (score 0–4), while the original CES-D was a 4-point Likert scale (score 0–3); consequently, the range of possible scores is between 0 and 80 in the CESD-R, and 0 and 60 in the CES-D. One of the most important reasons for this may be the reverse-scored items in the CES-D, as mentioned in the previous section. Thus, the meaning of the scores is naturally different, suggesting that the CES-D may not a good instrument by which to assess depression for epidemiological studies at present.

As mentioned in the introduction, more than forty years have passed since the publication of the original CES-D scale [12], and the CESD-R was undertaken to update the scale to reflect the DSM-IV text revision (TR) or DSM-5 classification [17]. This resulted in the creation of the CESD-R scale, consisting of 20 statements regarding well-being and behavior over the previous two weeks. The period of symptoms, which was extended according to DSM-IV-TR criteria from 7 days (CES-D) to 2 weeks (CESD-R), was compared to the CES-D scale.

### 4.5. Culture-Based Translation

There could be some problems in translation, because each Japanese item was selected as having back-translated English that was as close as possible to the original English item, without free translation, which made some expressions unnatural in the Japanese language and could make some questions difficult for participants to answer accurately. We tried to make various models, removing some items or increasing factors that lower the eigenvalue. Consequently, we think that the Japanese version used in the present study should be improved by assessing other populations or by changing the wording of the Japanese text. In addition, several items in the original English version may be inappropriate for non-English or non-European cultures. Some modification of items in translation and a cultural adaptation process for a measurement developed in English may be needed for the assessment to be applicable in the Japanese language or other cultures. Given the above considerations, we have displayed the modified Japanese translation in Table S3 for future studies.

### 4.6. Limitations

Participants in the current study were a small and localized sample, the validation of which was applied by explanatory factor analysis; confirmatory analysis will be needed in the future. The Japanese expression for each item might appear awkwardly phrased because we selected each Japanese expression to make as few differences in style (not in meaning) as possible between the original English text and the back-translated English text, without considering cultural issues influencing the wording.

## 5. Conclusions

The Japanese translation of the CESD-R scale may be used in population studies as a differentiating tool for the assessment of depressive symptoms. However, there are still some problems to solve regarding the wording of questions, as suggested by the revised version in Table S3. In addition, further investigation will be needed to assess the success of the questionnaire in other populations, applying confirmatory factor analysis.

## Figures and Tables

**Table 1:** Age and depressive degrees of participants (*n* = 398).

Variable	*n*	Mean ± S.D.	Range
Age	398	62.0 ± 10.36	41–90
Men	191	62.5 ± 10.45	41–89
Women	207	61.4 ± 10.27	41–90
Age	398	10.9 ± 6.63	0–42
Men	191	10.8 ± 6.34	0–38
Women	207	11.1 ± 6.90	0–42
Age	398	4.29 ± 6.52	0–42
Men	191	4.28 ± 6.37	0–42
Women	207	4.29 ± 6.67	0–36

CES-D: The Center for Epidemiologic Studies—Depression, CESD-R: CES-D—Revised, S.D.: standard deviation.

**Table 2:** Main factors of the CES-D and CESD-R scale.

**CES-D**			
**item**		**Factor 1**	**Factor 2**
6	I felt depressed.	0.79	
10	I felt fearful.	0.74	
18	I felt sad.	0.73	
7	I felt that everything I did was an effort.	0.73	
3	I felt that I could not shake off the blues, even with help from my family or friends.	0.69	
9	I thought my life had been a failure.	0.68	
1	I was bothered by things that usually don’t bother me.	0.68	
15	People were unfriendly.	0.67	
14	I felt lonely.	0.63	
19	I felt that people disliked me.	0.61	
5	I had trouble keeping my mind on what I was doing.	0.61	
17	I had crying spells.	0.61	
20	I could not get going.	0.57	
13	I talked less than usual.	0.56	
11	My sleep was restless.	0.52	
2	I did not feel like eating; my appetite was poor.	0.39	
8	I felt hopeful about the future.		0.77
12	I was happy.		0.75
4	I felt I was just as good as other people.		0.72
16	I enjoyed life.		0.67
**CESD-R**			
**item**		**Factor 1**	
13	I felt fidgety.	0.84	
4	I felt depressed.	0.83	
20	I could not focus on the important things.	0.77	
2	I could not shake off the blues.	0.77	
6	I felt sad.	0.74	
8	Nothing made me happy.	0.71	
21	I could not get going.	0.71	
3	I had trouble keeping my mind on what I was doing.	0.69	
17	I did not like myself.	0.68	
16	I was tired all the time.	0.66	
10	I lost interest in my usual activities.	0.59	
9	I felt like a bad person.	0.58	
5	My sleep was restless.	0.57	
14	I wished I were dead.	0.56	
12	I felt like I was moving too slowly.	0.55	
19	I had a lot of trouble getting to sleep.	0.52	
1	My appetite was poor.	0.32	
15	I wanted to hurt myself.	0.28	
11	I slept much more than usual.	0.19	
18	I lost a lot of weight without trying to.	0.12	

CES-D: The Center for Epidemiologic Studies Depression, CESD-R: CES-D—Revised.

## Data Availability

Non applicable.

## Supplementary Materials

The following are available online at https://www.mdpi.com/article/10.3390/bs11080107/s1, Table S1: the original English version of the CESD-R, Table S2: its Japanese version as analyzed in the current study, Table S3: The modified version of the Japanese CESD-R for future studies.
