# Supplementary material for: Validation of the Japanese Version of the Center for Epidemiologic Studies Depression Scale—Revised: A Preliminary Analysis
Source: Behav Sci (Basel). 2021 Jul 24;11(8):107. doi: 10.3390/bs11080107 (PMC8389265; doi:10.3390/bs11080107)
Supplement: Supplementary file 1 [file behavsci-11-00107-s001.zip › behavsci-1274507-supplementary.pdf]

Table S1. English version of CESD-R

Below is a list of the ways you might have felt or behaved. Please check the boxes to tell me how often you have felt this way in the past week or so.		Last Week				2 Nearly every day for 2 weeks
		Not at all or less than 1 day	1-2 days	3-4 days	5-7 days	
1	My appetite was poor.	0	1	2	3	4
2	I could not shake off the blues.	0	1	2	3	4
3	I had trouble keeping my mind on what I was doing.	0	1	2	3	4
4	I felt depressed.	0	1	2	3	4
5	My sleep was restless.	0	1	2	3	4
6	I felt sad.	0	1	2	3	4
7	I could not get going.	0	1	2	3	4
8	Nothing made me happy.	0	1	2	3	4
9	I felt like a bad person.	0	1	2	3	4
10	I lost interest in my usual activities.	0	1	2	3	4
11	I slept much more than usual.	0	1	2	3	4
12	I felt like I was moving too slowly.	0	1	2	3	4
13	I felt fidgety.	0	1	2	3	4
14	I wished I were dead.	0	1	2	3	4
15	I wanted to hurt myself.	0	1	2	3	4
16	I was tired all the time.	0	1	2	3	4
17	I did not like myself.	0	1	2	3	4
18	I lost a lot of weight without trying to.	0	1	2	3	4
19	I had a lot of trouble getting to sleep.	0	1	2	3	4
20	I could not focus on the important things.	0	1	2	3	4

Table S2. Japanese version of CESD-R used in the present study

以下に、あなたが感じたり、振る舞ったりした可能性のある事柄を挙げます。ここ2週間ほどで、自分自身に当てはまると感じた頻度について、右の選択肢から選んでください。		先週				2週間ほぼ毎日
		全くなし、または、1日未満	1〜2日	3〜4日	5〜7日	
1	食欲不振だった	0	1	2	3	4
2	憂うつな気分を拭い去ることができなかった	0	1	2	3	4
3	自分がしていることに集中できなかった	0	1	2	3	4
4	気分が落ち込んでいた	0	1	2	3	4
5	ぐっすり眠れなかった	0	1	2	3	4
6	悲しい気分だった	0	1	2	3	4
7	前に進めなかった	0	1	2	3	4
8	何についても、幸せを感じられなかった	0	1	2	3	4
9	自分自身が悪い人のような気がした	0	1	2	3	4
10	日常生活に関心をなくした	0	1	2	3	4
11	いつもよりずっと多く寝た	0	1	2	3	4
12	自分の動作が非常に緩慢だと感じた	0	1	2	3	4
13	気分が落ち着かなかった	0	1	2	3	4
14	死んでしまいたいと思った	0	1	2	3	4
15	自分自身を傷つけてしまいたかった	0	1	2	3	4
16	いつも疲れていた	0	1	2	3	4
17	自分自身のことが好きではなかった	0	1	2	3	4
18	努力したわけでもないのに、体重がかなり減った	0	1	2	3	4
19	寝付くのに苦労した	0	1	2	3	4
20	大事なことに集中できなかった	0	1	2	3	4

Table S3. Modified Japanese version of CESD-R for future studies

ここ2週間の心や体の状態についてお聞きいたします。<br>0〜4の当てはまるところに〇をつけてください。		先週				2週間<br>ほぼ毎日
		全くなし、または、1日未満	1〜2日	3〜4日	5〜7日	
1	食欲がない	0	1	2	3	4
2	気分が晴れない	0	1	2	3	4
3	何かと気が散る	0	1	2	3	4
4	憂うつだ	0	1	2	3	4
5	ぐっすり眠れない	0	1	2	3	4
6	なんとなく悲しい	0	1	2	3	4
7	なんだか億劫だ	0	1	2	3	4
8	何をしても楽しくない	0	1	2	3	4
9	自分は不道徳のような気がする	0	1	2	3	4
10	普段していた趣味などに、興味をもてない	0	1	2	3	4
11	いつもより、ずっと長く眠る	0	1	2	3	4
12	動作が緩慢になったようだ	0	1	2	3	4
13	イライラする	0	1	2	3	4
14	消えてしまいたい	0	1	2	3	4
15	自分を責めてしまう	0	1	2	3	4
16	ずっと疲れている	0	1	2	3	4
17	自分のことが好きでない	0	1	2	3	4
18	ダイエットしていないのに、体重が減った	0	1	2	3	4
19	なかなか寝付けない	0	1	2	3	4
20	物事に集中できない	0	1	2	3	4
